# Implementation of a Patient Navigation Program to Support Representative Participation in Cancer Clinical Trials

**DOI:** 10.1002/cam4.71125

**Published:** 2025-08-02

**Authors:** Sukh Makhnoon, Fabian Robles, Jessica L. Lee, D'Angelo Grant, Marisol Rojas, Ang Gao, Carla Pezzia, Venkata Eluri, Navid Sadeghi, Song Zhang, Thomas Hulsey, Rebecca Renn, Erin L. Williams, Heather Kitzman, David E. Gerber

**Affiliations:** ^1^ Harold C. Simmons Comprehensive Cancer Center UT Southwestern Medical Center Dallas Texas USA; ^2^ O'Donnell School of Public Health UT Southwestern Medical Center Dallas Texas USA; ^3^ Parkland Health Dallas Texas USA; ^4^ Biology Department University of Dallas Irving Texas USA; ^5^ Division of Hematology‐Oncology, Department of Internal Medicine UT Southwestern Medical Center Dallas Texas USA

**Keywords:** cancer, clinical trials, diversity, implementation science, patient navigation

## Abstract

**Background:**

Achieving adequate, timely, and diverse trial enrollment remains a major challenge in clinical research. Insufficiently diverse patient representation compromises the generalizability of clinical trial findings and remains a persistent issue in oncology. Navigation services may help patients learn about clinical trials, identify and overcome barriers, and progress through the care pathway to trial enrollment and retention.

**Methods:**

We implemented a patient navigation program to support diverse enrollment and retention of patients in cancer clinical trials; the proximal outcomes were receipt of financial navigation and trial interest. The study was conducted from July 2023 to July 2024 at two demographically diverse health care settings: a university‐based tertiary healthcare system and an integrated safety‐net healthcare system. Evaluation was guided by the Reach, Effectiveness, Adoption, Implementation, and Maintenance framework and incorporated programmatic data, structured surveys of patients and staff, and qualitative patient interviews.

**Results:**

The program navigated 429 oncology patients (52% female, 28% Hispanic/Latino (HL), and 16% non‐HL Black). Compared to the underlying patient population of the clinical settings, program participants were more likely to be Hispanic (31% vs. 21%; *p* < 0.01), female (52% vs. 48%; *p* = 0.01) and from a minority race (30% vs. 24%, *p* ≤ 0.01). Within the population who were successfully contacted, 325 of 408 (92%) patients already enrolled in a trial received financial navigation to help with trial retention. Among the remaining 83 patients not enrolled in a cancer clinical trial at the time of referral, 39 (47%) expressed interest in participating in a clinical trial in thefuture.

**Conclusion:**

A patient navigation program to influence enrollment and retention of diverse patients into trials was feasible to implement, highly acceptable to patients, and reached a priority population of patients generally underrepresented in cancer clinical trials. Further research into the effect of navigation on trial enrollment and retention is warranted.

## 1 Introduction

Insufficiently diverse patient representation compromises the generalizability of clinical trial findings and remains a persistent issue in oncology [1]. Achieving diverse enrollment may reduce the efficacy‐effectiveness gap (i.e., outcome differences between patients treated in trials and those in clinical practice) [2] and identify pharmacologic, efficacy, and safety differences across populations [3]. Yet despite much attention to the issue, there remain few evidence‐based approaches that increase accrual diversity [4]. One approach, navigation services, may help patients learn about cancer clinical trials, identify and overcome barriers, and progress through the care pathway to enrollment and retention [5, 6].

Patient navigation has been implemented across the cancer care continuum, from screening and prevention to coordination of multi‐modality therapy. Navigation programs increase cancer screening rates, treatment adherence, quality of life, and satisfaction with care, improve cancer outcomes, and reduce costs [7]. Recent years have also seen the advent of navigation programs focused on cancer clinical trial enrollment [8]. However, to date the published experience with this strategy is limited to studies focusing on a single demographic group (e.g., Black individuals) with some also addressing only a single cancer type (e.g., breast cancer) [9, 10, 11]. Thus, it is unclear if findings are generalizable to other populations or other malignancies.

Development of navigation programs for cancer clinical trials or cancer care more broadly represents a complex process and requires detailed attention to institutional and regional needs. Yet few reports have focused on how such interventions are implemented, adapted over time, evaluated, and sustained [5, 12]. We therefore implemented a navigation program across all cancer types to support representative trial enrollment at the two main clinical sites of a National Cancer Institute‐designated comprehensive cancer center: a university‐based tertiary healthcare system and an integrated safety‐net healthcare system. Providing care for a demographically and socioeconomically diverse patient population, the settings provide an opportunity to implement and evaluate navigation. Here we evaluate program implementation, acceptability, reach, effectiveness, and maintenance across multiple domains.

## 2 Methods

We invited a subset of systematically selected navigation program patients (every *n*th patient) to participate in a program evaluation study. Study procedures were approved by the University of Texas Southwestern Institutional Review Board and by the Parkland Health Office of Research Administration (STU‐2021‐1144). All survey respondents and interviewees provided informed consent in their preferred language. Given the program improvement goals and large sample size of participants, we received a waiver of consent to collect medical record information. We followed the Standards for Reporting Quality Improvement Reporting Excellence (SQUARE2.0) guidelines [13].

### 2.1 Setting

We implemented the navigation program at the UT Southwestern Harold C. Simmons Comprehensive Cancer Center (SCCC), which provides adult clinical care at a university hospital and outpatient clinics (UT Southwestern Medical Center [UT Southwestern]) and an integrated safety‐net healthcare system (Parkland Health [Parkland]) in Dallas, Texas. Dallas County has a population of 2.6 million (42% Hispanic, 22% Black), of whom 14% live in poverty and 21% lack health care coverage [14]. Parkland includes 12 community‐based primary care clinics located throughout Dallas County and a main campus located less than one mile from the UT Southwestern outpatient cancer clinics. Cancer care at both Parkland and UT Southwestern is provided by UT Southwestern faculty physicians and post‐doctoral fellows who are organized into multidisciplinary oncology teams according to cancer type: brain/CNS, breast, gastrointestinal, genitourinary, gynecologic, head and neck, malignant hematology, lung, and melanoma/skin/sarcoma. Similarly, SCCC Clinical Research Office personnel (UT Southwestern employees) staff both sites. Because the program and this study focused on adults with cancer, we did not develop the navigation program at Children's Health, the SCCC affiliate that provides pediatric cancer care.

### 2.2 Evidence‐Based Intervention Components and Implementation Strategies

The navigation program is a multicomponent evidence‐based intervention designed to identify oncology patients who might benefit from and provide dedicated support services, including education, coordination, and reimbursement for nonmedical trial‐related costs for patients who earn below 700% of the federal poverty level [15]. The financial navigation and resource addresses “financial toxicity”—a term that encompasses burdens and concerns related to affordability—that patients may face when considering trials. These include additional nonmedical out‐of‐pocket costs, more frequent visits at more distant clinical sites, and more missed workdays, which in turn may contribute to the lower rates of trial participation among lower‐income individuals [16, 17, 18, 19, 20]. Four dedicated oncology patient navigators, two at each site, worked within the healthcare systems to provide individualized assistance to patients.

We intended the program to integrate into existing workflows, supporting early identification of patients who may need support enrolling or retaining in trials, then referring them for navigation to receive educational materials and resources for trial enrollment and retention. Each healthcare system customized the program to suit workflow and needs. At Parkland, nurse managers refer patients for trials, at which point navigators are notified. At UT Southwestern, patients self‐report interest in trials, triggering navigator notification. At both sites, clinicians, clinic staff, and clinical research personnel can refer patients as well.

Based on the Expert Recommendations for Implementation Change [21], we identified strategies a priori to promote equitable access and uptake of the program at each site. Based on stakeholder feedback, we tailored approaches, including: (1) creating new educational materials in English and Spanish, (2) colocating navigators with other clinical research office staff (managers, nurses, coordinators, data specialists), (3) conducting educational meetings with clinical stakeholders, (4) creating new clinical workflows, (5) developing a quality monitoring system, and (6) leveraging clinic champions (Data S1).

### 2.3 Theoretical Framework for Evaluation

We evaluated the patient navigation program according to the Reach, Effectiveness, Adoption, Implementation, and Maintenance (RE‐AIM) framework [22]. Table 1 describes our operationalization of the RE‐AIM constructs. Data used to support these constructs included observational data of ongoing program implementation (navigation contacts, content, and outcome), facility‐level clinical and demographic data (information from electronic medical record), programmatic data, and patient‐reported data (qualitative data on experience with navigation).

**TABLE 1:** Operationalization of the reach, effectiveness, adoption, implementation, and maintenance (RE‐AIM) framework for the clinical trial navigation program.

RE‐AIM construct	Measures of navigation program	Data source(s)
Reach	Proportion of oncology patients who participate in the program	EMR
	Proportion of underrepresented oncology patients who participate in the program	Cancer registry
	Demographic and cancer characteristics	Program database
Effectiveness	Proportion of patients who express interest in participating in a cancer clinical trial	REDCap screening and program database
Adoption	Setting level characteristics: No. of oncology teams who have referred pts. to the program	REDCap referral log
	Staff level characteristics: No. of clinicians and team members and their roles	
Implementation	Acceptability—patient reported experience with program participation	Patient interview data
	Program fidelity—use of the core components of navigation	Encounter summaries documented in REDCap database
Maintenance	Sustainability of navigation program	PNSAT sustainability survey data
	Sufficiency of number and type of staff to provide navigation	Summary data from program investigator meetings

Abbreviations: EMR, electronic medical record; PNSAT, Patient Navigation Sustainability Assessment Tool; REDCap, Research Electronic Data Capture.

#### 2.3.1 Reach

To determine the number and characteristics of oncology patients identified and reached through the program, we quantified the number of unique patients referred to the program during the implementation period using patient clinical and demographic data. For the year of program implementation, we summarized cancer registry information to identify (1) the number of incident cancer cases at each site; (2) patients' sex, race, and ethnicity; and (3) the proportion of the population of interest (i.e., underrepresented in cancer clinical trials [female; age ≥ 65 years; racial/ethnic minority] [23, 24, 25]) that the navigation program was able to reach.

#### 2.3.2 Effectiveness

The co‐primary effectiveness outcomes of the navigation program were operationalized as (1) the percentage of navigated who were assisted with financial navigation and (2) the patients who expressed interest in trial participation. At this stage, we did not select clinical trial enrollment among navigated patients as the effectiveness outcome because numerous factors beyond patient interest and access (e.g., trial availability, eligibility criteria) can affect enrollment.

#### 2.3.3 Adoption

For each program site, we collected information on clinic organization and staff (number and role [trainee or faculty] of clinicians). To understand program adoption, we analyzed referrals received through each oncology team relative to the number of patients seen by the team.

#### 2.3.4 Implementation

We selected implementation outcomes to assess program acceptability and fidelity. Program participants were invited to participate in a qualitative interview about their navigation experience. We measured the acceptability of the navigation program among patients using qualitative data from patient interviews. We evaluated program fidelity (i.e., the extent to which core components of navigation were used) by analyzing patient‐navigator encounters recorded in a Redcap database. We also recorded adaptations made to the navigation program after its implementation.

#### 2.3.5 Maintenance

At both sites, we distributed surveys to clinical and administrative leadership involved with program implementation to determine the extent to which the navigation program became integrated within the institutions' organizational practices and policies. Surveys assessed program sustainability using items from the Patient Navigation Sustainability Assessment Tool (PNSAT) [26], which scores a set of eight program characteristics (e.g., engaged staff and leadership, funding stability), each on a scale of 1 (“program has this to little or no extent”) to 7 (“program has this to a great extent”).

### 2.4 Data Analysis

#### 2.4.1 Quantitative Data

Descriptive statistics were reported, including means, standard deviations, medians, and inter‐quartile ranges (IQRs) for continuous variables, and counts and percentages for categorical variables. Comparisons between groups based on the Chi‐square test were performed. All statistical analyses were conducted using SAS 9.4 (SAS Institute, Cary, NC).

#### 2.4.2 Qualitative Data

We audio recorded, transcribed, and conducted rapid qualitative analysis of semi‐structured 1:1 patient interviews to generate actionable targeted data to guide program improvement [27, 28]. We created a template for summarizing data, populated it using transcripts, and synthesized results. Quotations illustrating the main themes were identified during the process of rapid analysis.

Navigators recorded a summary of each patient‐navigator encounter in an open‐ended Redcap field. We used inductive thematic analysis to analyze these free text responses. One qualitative analyst (RR) independently developed the codebook and coded a subset of the dataset to develop categories emerging from the codes. A second qualitative analyst (JL) re‐coded the data using the previously developed codebook. Discrepancies were resolved by the analysts with input from the senior arbitrator (SM).

## 3 Results

Between July 2023 and July 2024, 429 oncology patients were referred to the navigation program (298 at UT Southwestern, 131 at Parkland). Patients had a median age of 62 years (IQR 51–69 years), 52% were female, 31% were Hispanic, and 16% were non‐Hispanic Black. Additional characteristics of program participants are shown in Table 2.

**TABLE 2:** Sociodemographic characteristics of patients referred to the navigation program (*n* = 429).

Variable	Categories	UT Southwestern (*N* = 298) Median (IQR) or *n* (%)	Parkland (*N* = 131) Median (IQR) or *n* (%)	Total *N* = 429 Median (IQR) or *n* (%)
Age, years		64 (55–72)	55 (49–63)	62 (51–69)
Sex	Female	116 (39)	102 (81)	218 (52)
	Male	181 (61)	24 (19)	205 (49)
Preferred language	English	270 (92)	42 (32)	312 (73)
	Spanish	24 (8)	88 (67)	112 (26)
Race/Ethnicity	White Hispanic/Latino	36 (12)	83 (64)	119 (28)
	Asian not Hispanic/Latino	17 (6)	4 (3)	21 (5)
	Black not Hispanic/Latino	37 (12)	31 (24)	68 (16)
	White not Hispanic/Latino	172 (58)	8 (6)	180 (42)
	Other	36 (12)	3 (2)	39 (9)
Marital status	Single	63 (21)	44 (34)	107 (25)
	Married	177 (58)	48 (38)	225 (53)
	Separated or divorced	22 (7)	24 (19)	46 (11)
	Widowed/Lives with partner/Declined to answer	34 (11)	12 (9)	46 (11)
Insurance coverage (multiple allowed)	Medicaid/Managed care	6 (2)	6 (4)	12 (3)
	County health plan	14 (5)	80 (61)	94 (22)
	Private/Commercial	154 (52)	5 (3)	159 (37)
	Medicare‐standard	67 (23)	10 (7)	77 (18)
	Medicare advantage	48 (16)	7 (5)	55 (13)
	Other/Unknown	10 (4)	17 (14)	27 (6)
Employment status	Full‐time	106 (36)	27 (26)	133 (34)
	Unemployed	66 (23)	49 (47)	115 (29)
	Retired	110 (38)	13 (13)	123 (31)
	Part‐time/Disabled	10 (3)	15 (15)	25 (7)
Family income sources (multiple allowed)	Social security (retirement)	125 (42)	9 (7)	134 (31)
	Salary/Wages	111 (37)	33 (25)	144 (34)
	SSDI or SSI/Unemployment/Public Assistance	13 (5)	10 (8)	23 (4)
	Family/Friends	21 (7)	36 (28)	57 (13)
Cancer type	Brain/Central nervous system	3 (1)	5 (4)	8 (2)
	Breast	38 (13)	70 (53)	108 (25)
	Gastrointestinal	34 (11)	14 (11)	48 (11)
	Genitourinary	123 (41)	8 (6)	131 (31)
	Gynecological	3 (1)	14 (11)	17 (4)
	Head and neck	41 (4)	0 (0)	41 (10)
	Malignant hematology	16 (5)	0 (0)	16 (4)
	Lung	18 (6)	3 (2)	21 (5)
	Melanoma/Skin/Sarcoma	4 (1)	0 (0)	4 (1)
	Other	14 (5)	1 (1)	15 (4)
	Missing	4 (1)	16 (12)	20 (4)
Enrolled in a cancer clinical trial at referral	No	26 (9)	78 (67)	104 (25)
	Yes	268 (92)	39 (33)	307 (75)

### 3.1 Reach

Table 3 shows program reach by clinical site and patient demographics. As intended, the program reached patients underrepresented in cancer clinical trials. Compared to the underlying population, program patients were more likely to be Hispanic (31% vs. 21%; *p* ≤ 0.01), from a minority race (30% vs. 24%, *p* < 0.01), and aged > 65 years (44% vs. 38%; *p* = 0.01). Overall, 378 of 429 (88%) navigation patients were from underrepresented populations. With a total of 8144 cancer cases among underrepresented patients across both clinical sites (3937 enrolled in cancer clinical trials), the navigation program reached approximately 5% of this population of interest (and 12% of the population of interest enrolled in trials).

**TABLE 3:** Characteristics of program participants compared to overall clinic population based on newly diagnosed cancer cases in 2023.

	UT Southwestern			Parkland			Total		
	Program (*N* = 298) *n* (%)	Population^a^ (*N* = 7201) *n* (%)	*p*	Program (*n* = 131) *n* (%)	Population^a^ (*N* = 2255) *n* (%)	*p*	Program (*N* = 429) *n* (%)	Population^a^ (*N* = 9774) *n* (%)	*p*
Minority race^b^	89 (30)	1584 (22)	< 0.01	38 (29)	721 (32)	0.52	128 (30)	2345 (24)	< 0.01
Hispanic	49 (16)	792 (11)	< 0.01	83 (65)	1262 (56)	0.04	132 (31)	2052 (21)	< 0.01
Female sex	116 (39)	3240 (45)	0.04	102 (81)	1307 (58)	< 0.01	218 (52)	4691 (48)	0.14
65 years or older	143 (48)	3600 (50)	0.44	21 (16)	541 (24)	0.03	163 (38)	4202 (43)	0.01

^a^ Cancer registry data.

^b^ Includes patients identifying as African American or Black; American Indian or Alaskan Native; Asian or Native Hawaiian or Pacific Islander.

### 3.2 Effectiveness

Of the 429 referred patients for navigation services, 408 (95%) were successfully contacted. Navigation discussions generally focused on (a) opportunities for financial reimbursement of nonmedical, trial‐related costs and/or (b) clinical trial participation. Among navigation recipients, 325 (92%) patients received financial navigation to help with enrollment and retention. Among the remaining 83 patients not enrolled in a trial at referral, programmatic data showed that 39 (47%) expressed interest in participating in a trial, 15 (18%) expressed interest in learning more about trial participation, and 7 (8%) were not interested in participating in a trial. The remaining 17 (21%) patients screen‐failed for trials and 5 (6%) enrolled in a nontreatment trial.

### 3.3 Adoption

**FIGURE 1:**
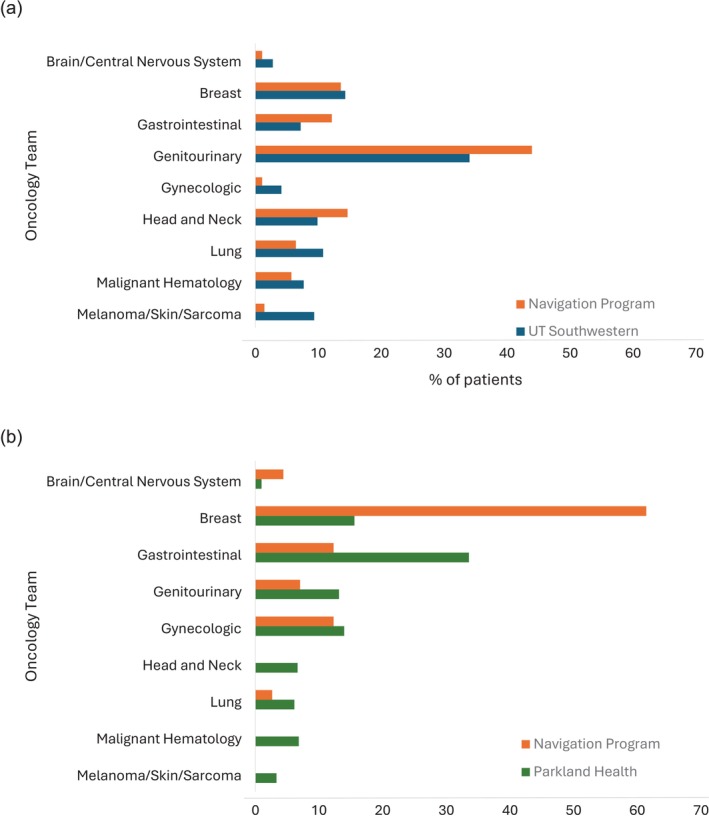
Adoption of navigation program by oncology teams at the two study sites. Proportion of patients referred to the navigation program is compared to oncology teams' relative patient volumes at (a) UT Southwestern and (b) Parkland.

At both UT Southwestern and Parkland, all approached oncology teams (*n* = 9) referred patients to the navigation program. Across teams, referral volumes generally corresponded to the team's patient volumes. Compared to other oncology teams, referral numbers were relatively higher for the genitourinary team and lower for the gynecologic and melanoma/skin/sarcoma teams at UT Southwestern (Figure 1). We recorded a patient's primary oncologist as the referring provider, although in practice research coordinators, nurses, and advanced practice providers could initiate the referral process. Participation as a proportion of specialists practicing at the two clinical sites was as follows: hematology‐oncology, 48/87 (55%); radiation oncology, 14/25 (56%); gynecologic oncology, 4/13 (31%); surgical oncology, 2/27 (7%); a hepatologist and a urologist.

### 3.4 Implementation

After program initiation, each clinical site made a variety of adjustments to maximize navigation uptake and effectiveness. At UT Southwestern, the financial navigation component of the program was implemented before the component focused more broadly on trial enrollment. As a result, clinic and research staff had limited awareness of general navigation services when they were rolled out. Navigators addressed this issue through additional outreach and education about general navigation services. At Parkland, where post‐doctoral clinical oncology fellows under faculty supervision serve as the primary oncologists for many patients, navigators observed that the time and effort required for these trainees to complete and document clinical care hindered consideration of clinical trials and related navigation services. Navigators addressed this concern through active and repeated engagement with rotating fellows and supervising faculty, demonstrating that enrollment in trials and referral for associated navigation might not only elevate the level of care provided, but also improve long‐term workflows.

We used qualitative methods to assess patient acceptance of the navigation program and perceived utility of navigator coordination efforts. Eleven program participants were invited to participate in qualitative interviews, all of whom agreed to participate. Overall, patients reported that their experience with the navigator was “very good.” Patients appreciated interacting with navigators, both in‐person and via telephone, during their care, “[navigator] came to meet us twice when we were down there to facilitate turning in the forms and things. She was just, she was very accommodating and very helpful, very knowledgeable.”

#### 3.4.1 Fidelity

Four patient navigators made 1339 contact attempts as part of the program with an average of two contact attempts per patient (range 1–16) via telephone (71%), in person (24%), and using a secure electronic patient portal (MyChart, 5%). Of the 698 unique documented encounters between a patient and a navigator, detailed encounter summaries were available for 595 (85%). These interactions included discussions of financial reimbursement for nonmedical costs related to trial participation (including interest in, eligibility for, and assistance with the program), conversations about interest in clinical trial participation, assessment of barriers, and education about clinical trials.

### 3.5 Maintenance

**FIGURE 2:**
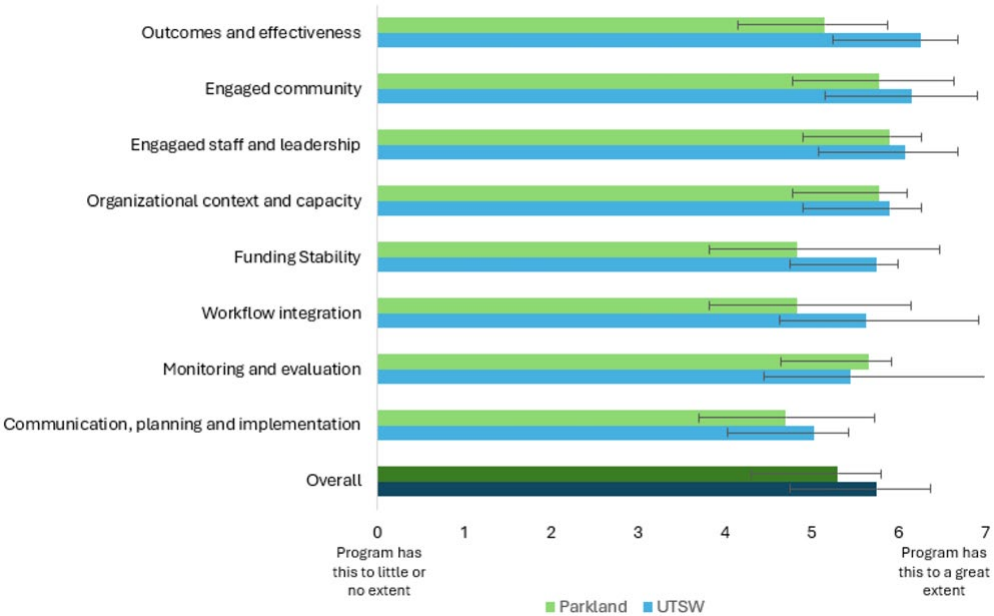
Sustainability assessment of the patient navigation program. Patient Navigation Sustainability Assessment Tool (PNSAT) score distribution.

Seven stakeholders representing a variety of roles involved in program implementation (e.g., navigator supervisors, clinical leadership, financial leadership) completed the PNSAT survey. The mean (± standard deviation) program sustainability score (out of 7) was 5.5 ± 0.60 across both institutions (5.3 ± 0.5 at UT Southwestern 5.8 ± 0.6 at Parkland). Program staff and leadership engagement received the highest scores (mean 6.0 ± 0.5), whereas communication, planning, and implementation received the lowest scores (mean 4.8 ± 0.8). Distribution of scores for each program characteristic and institution is in Figure 2.

## 4 Discussion

We report the implementation outcomes of a clinical trial navigation program that served patients across cancer types, populations, and clinical settings. Because eventual clinical trial enrollment reflects numerous trial‐ and setting‐specific factors that may not be modified by navigation (e.g., trial availability, eligibility criteria), in this study we focused on proximal outcomes (receipt of navigation, patient interest in trial participation) potentially influenced by navigation. We found that the navigation program was feasible to implement, highly acceptable to patients, and reached our priority population of patients underrepresented in cancer clinical trials. Over a 1‐year period, the program provided navigation services to more than 400 patients with cancer, of whom almost 90% were historically underrepresented in cancer clinical trials. Compared to prior work where 445 patients were navigated over 5 years (78% Caucasian) [29], this program navigated a similar volume of and more diverse patients in 1 year. About three‐quarters of navigation recipients had already been enrolled in a trial, in which case the program provided information and access to financial reimbursement for out‐of‐pocket nonmedical costs to enhance trial retention. The remaining patients received navigation prior to the point of potential trial enrollment and received education, guidance, and support through trial consideration and screening. The program identified patient interest in trial participation, had broad and equitable reach in facilitating trial retention, and was felt to be sustainable. These achievements were supported by infrastructure, financial resources, and a staff of multiple patient navigators, some of whom were bilingual.

As a marker of program adoption, we tracked the number of patient referrals according to cancer type. Although almost all major cancer types were represented, referral numbers were not necessarily proportional to patient volumes. Potential reasons for this variability include differences in the number of available clinical trials, staff buy‐in, and navigator interactions. For instance, at Parkland, a large number of underrepresented minority patients with gynecologic malignancies or prostate cancer, coupled with activated trials for those conditions, led clinical research program managers in those areas to encourage their staff to incorporate navigation referrals into their regular workflows. Also at Parkland, the breast cancer program implemented a separate patient navigation program for routine clinical care, which in turn made referrals for clinical trials navigation a logical and straightforward step. In our experience, stakeholder engagement prior to and throughout program implementation was crucial to program success. In the future, education of clinicians and clinic staff may help clarify the professional responsibilities of clinical trial patient navigators and distinguish their roles from those of study coordinators, social workers, and other clinic staff. Explicit definitions of patient navigation, such as that proposed by the American Cancer Society, may help in these efforts and preemptively address concerns of staffing redundancy [30].

Future continuation of the navigation program remains a key focus, as the eventual goal is to transition from extramural grant funding to institutional support. In addition to the recent Medicare funding for patient navigation under the Cancer Moonshot Program, our sustainability planning process identified engaged staff and leadership as domains that may support the program's continued implementation. Communication, planning, and implementation were identified as domains with room for improvement, especially in terms of streamlining clinical workflow and patient referrals. We are planning an analysis of cost and cost‐effectiveness, which together with the new Center of Medicare and Medicaid Services rule to bill for patient navigation services may make an economic case for program sustainability and institutional support [31]. Public awareness campaigns targeting physicians and clinical staff will help ensure continued demand and referrals to the program.

The study has a number of limitations. First, implementation outcomes from an academic medical center and its affiliated urban safety‐net site may not be generalizable to other community‐based oncology facilities serving more rural populations. Second, there may be potential for self‐selection bias, as oncology teams that referred patients for navigation may be more motivated to make changes in representative trial enrollment and thus engage with the program. Nevertheless, the program received referrals from all oncology teams, demonstrating high reach. Third, acceptability data from oncology clinic staff was not available. To address this limitation, navigators colocated with clinic staff routinely solicited and conveyed staff feedback at program team meetings. Fourth, a proportion of our program patients were already enrolled in a trial and received financial support for trial retention; such resources may not be available at other centers. Finally, data on certain confounders (e.g., organizational readiness for change) was not available. Key strengths of the study include the prospective and longitudinal collection of programmatic data, diversity of clinical settings and patient population, and programmatic evaluation using a rigorous implementation science framework.

In conclusion, we found that a patient navigation program to support representative enrollment and retention of patients into cancer clinical trials could be implemented and widely adopted, achieving broad and equitable reach, and demonstrating preliminary effectiveness. Patient navigation that addresses available trials and financial support represents a viable approach to increasing trial enrollment and retention. It is hoped that the present experience provides valuable lessons for future adoption and implementation of navigation services within other healthcare organizations.

## Author Contributions

**Sukh Makhnoon:** writing – review and editing, writing – original draft, methodology. **Fabian Robles:** project administration, writing – review and editing. **Jessica L. Lee:** project administration, writing – review and editing, data curation. **D'Angelo Grant:** project administration, writing – review and editing. **Marisol Rojas:** project administration, writing – review and editing. **Ang Gao:** data curation, formal analysis, writing – review and editing. **Carla Pezzia:** writing – review and editing, methodology, project administration. **Venkata Eluri:** writing – review and editing, project administration. **Navid Sadeghi:** investigation, supervision, writing – review and editing. **Song Zhang:** formal analysis, supervision, writing – review and editing. **Thomas Hulsey:** project administration, writing – review and editing. **Rebecca Renn:** formal analysis, data curation, writing – review and editing. **Erin L. Williams:** supervision, investigation, writing – review and editing. **Heather Kitzman:** writing – review and editing, methodology. **David E. Gerber:** conceptualization, funding acquisition, project administration, resources, writing – review and editing.

## Conflicts of Interest

The authors report no disclosures related to this work. Beyond the scope of this work, D.E.G. reports consulting fees from Catalyst Pharmaceuticals; U.S. patent 11,747,345; pending patents 17/045,482, 18/504,868, 63/386,387, 63/382,972, and 63/382,257; research funding from AstraZeneca, Karyopharm, and Novocure; participating in advisory boards for Astra‐Zeneca, Daiichi‐Sankyo, Elevation Oncology, GSK, Janssen Scientific Affairs, Jazz Pharmaceuticals, Regeneron Pharmaceuticals, Sanofi, and Summit Therapeutics; stock shares in Gilead; and serving as cofounder and Chief Medical Officer of OncoSeer Diagnostics Inc.

## Supporting information


**Data S1:** Intervention components, functions, and implementation strategies.

## Data Availability

The data that support the findings of this study are available from the corresponding author upon reasonable request.
