# Supplementary material for: Implementation of a Patient Navigation Program to Support Representative Participation in Cancer Clinical Trials
Source: Cancer Med. 2025 Aug 2;14(15):e71125. doi: 10.1002/cam4.71125 (PMC12317281; doi:10.1002/cam4.71125)
Supplement: Supplementary file 1 — Data S1: Intervention components, functions, and implementation strategies. [file CAM4-14-e71125-s001.docx]

**Supplementary Information**

**Supplement 1.** Intervention components, functions, and implementation strategies.

| **Intervention components and functions** | **Implementation strategies and program operationalization** |
| --- | --- |
| Identify patients who might benefit from support to participate in cancer clinical trials   - Being considered for trial enrollment or have already enrolled into a trial, or - Identify as an underrepresented patient population in trials (racial/ethnic minority, low income, under or uninsured)   Coordinate care for identified patients through navigation services   - Establish relationships with oncology clinicians, clinic staff, and clinical research personnel - Facilitate appointment scheduling between oncology clinic and research teams - Track appointments and results   Provide access to financial, logistical, and support services   - Financial and insurance resources - Transportation, childcare, and community resources - Education about clinical trials | Identify champions  Recruit staff, clinicians, and other system stakeholders to support implementation  Create new clinical workflows  Develop new communication processes between oncology clinical personnel, navigators, and clinical research office staff to improve care coordination and trial enrollment  Conduct educational meetings  Educational meetings with oncology team staff for program awareness and engagement.  Develop quality monitoring system  Create screening and program database to improve performance evaluation and programmatic decision making |
